# Symptoms of Social Disorganization*Read before the San Antonio Scientific Society, January 12, 1915.

**Published:** 1915-02

**Authors:** Theo. Y. Hull


### The Department of Eugenics, Mental and Social Hygiene

CONDUCTED BY
DR. MALONE DUGGAN
813-814 Gibbs Building, San Antonio, Texas

All communications for this department should be sent to DR. DUGGAN

### Symptoms of Social Disorganization.*

BY THEO. Y. HULL, M. D.

In the course of social evolution many conditions arise which, in time, if not arrested, would destroy society itself, or reduce it again to the state of barbarism or even savagery from which it sprang. If the three great plagues, alcoholism, tuberculosis, and the social diseases, recognized as universal in their evil effects, were permitted to continue their ravages unchecked, it would be only a question of time when racial destruction would occur. Fortunately, whenever an evil condition seriously menaces the social structure or the racial integrity, the very condition itself excites remedial action. Thus, abuse of power or privilege excites reaction. Sometimes, as in the case of ancient Greece and early Rome, the reaction does not occur at a sufficiently early stage to prevent serious racial degeneration. The period at which this awakening does occur, and the intensity of the movement, indicate the state of social development. In the twenty centuries or more since Greece and Rome were at the height of their national achievements, the social condition of the masses, the average lot has changed greatly for the better. Where a few raised their voices in denunciation of the evils of that day, the many now seek to assist in directing the course of social reform. There exists an inherent tendency to recover from dangerous conditions, and this tendency in the social body gives rise to optimism at times when pessimism might find ample nourishment.

But social reform is often bought at too high a price. The conditions that are symptomatic of social disorder are not imaginary. They are real. Those who point out social conditions are not alarmists. President Wilson has said that "a nation is a living thing and not a machine." Likewise a people is composed of individuals which cannot be treated as a group. There are those

*Read before the San Antonio Scientific Society, January 12, 1915.

who are living far ahead of the average life. There are also those who still linger in the past. But when a downward evolution threatens the social body, the symptoms must be brought home to the average conception. War often results from the failure to detect and check unfavorable tendencies. Mere tendencies at one time become fixed purposes at another. A titanic struggle ensues. Human life is sacrificed, and the evil corrected, but at a terrible cost.

There are three symptoms of the social malady that I wish to discuss tonight. These are (a) mental unbalance, (b) physical disease, and (c) a declining birth rate. Mental unbalance is not all seen in the asylums for the insane. Dr. Godding, the head of one of the largest asylums in this country, said that these institutions contained but a small per cent of the mentally unbalanced. Insanity is but one manifestation of mental instability. Pauperism, feeble-mindedness and crime are manifestations of mental weakness which differ largely in degree only. Pauperism is probably one of the mildest forms of mental unbalance or weakness. With the ever increasing complexity of life the struggle for existence becomes more and more intense. In earlier days many individuals possessed of sufficient physical strength were capable of self-support without burdening the community, but with greater competition these are thrust aside as unable to continue the struggle, and become charges either upon the State or the community. At the close of 1910 336,127 persons were inmates of benevolent institutions in this country. During the same year approximately 3,000,000 persons were admitted to the same institutions, and nearly 2,500,000 more were treated in free dispensaries. In other words, during the year 1910 5.87 per cent of the population, or one in every seventeen persons, were objects of either public or private charity. What is the significance of this condition? Two simple facts may aid in drawing an inference. In 1890, 34.4 per cent of the people owned unencumbered homes; in 1900, the percentage had decreased to 31.7, and in 1910, to 30.8 per cent, showing a steady decline in financial independence. In 1890, 37.6 per cent of the people lived on farms, the most independent life possible. In 1900, 35.2 per cent occupied farms, while in 1910, 30.2 per cent were farmers. These two facts show the tendency of the American people to drift from the simpler to the more complex conditions of life. The stronger members are successful, while weaker fail continually and are thrust back upon society as incapable of continuing an independent and unaided existence, and become partially or entirely objects of charity.

Feeble-mindedness marks a still greater departure from the normal. While pauperism may indicate a lack of power for the initiative or an inability to secure consecutive action, feeble-mindedness indicates a deficiency of mind. Dr. Goddard defines the feeble-mindedness as individuals "who cannot compete on equal terms with their fellows or manage their own affairs with ordinary prudence." In other words, such persons "are capable of living an independent existence, but must be dependent upon others." In 1890 there were 20,731 feeble-minded persons in institutions provided for their care. Of these 93.5 per cent were in public institutions. Those kept in such institutions represent usually the extreme cases. The number so cared for represents but a small per cent of the actual number of the mentally deficient. Dr. Goddard referred to above says that "it is a conservative statement to declare that 2 per cent of public school children are distinctly feeble-minded." If this statement is correct, there are today in this country approximately 400,000 mentally deficient children of school age, and how many others of the same class we have no means of knowing accurately. These constitute not only a burden upon the public, but, in view of our weak and defenseless marriage customs, they constitute a more serious problem for the future. Aside from being merely weak of mind, this class contributes largely to pauperism, crime and disease and insanity. Under the present system, or want of system, in dealing with this class, who are mentally unfit to care for themselves, the condition must unavoidably grow worse. It must be a potent factor in national and racial degeneracy.

While we class pauperism and feeble-mindedness as varying degrees of mental weakness and mental deficiency, crime represents a mental perversion. It is an abnormality. Man is not naturally a criminal, notwithstanding the theological doctrine of natural depravity to the contrary. The distorted view that such a person holds of his moral relations to others is just as much an evidence of an abnormal mental condition as insanity. But crime, like poverty, is always with us, and will be. What concerns us most, however, is whether there is a relative and progressive increase in crime and the number of criminals. Referring again to the census for 1910, we find that the number of prisoners confined in penitentiaries, jails and workhouses January 1, 1910, was 111,498, and that during the year there were committed to the same institutions 479,787. The year 1910 was in no sense an abnormal year; that is, there were no unusual conditions calling for more than the usual degree of self-control. The number committed during the year represents the average crop of criminals convicted. The number barely escaping conviction we have no means of knowing. Add to this class the annual crops of juvenile delinquents, 14,147 for the year 1910 (24,974 in institutions January 1, 1910), and we have an aggregate number of about one-half a million persons of this type of mental unbalance convicted of crimes ranging from petty stealings to the gravest murder. The presence of such an army of individuals with low moral conception is demoralizing. Statistics show that the condition is increasing. In 1850, when life was much less complex, there were less than 7000 prisoners in the United States. During the following fifty-four years the number increased fifteen times. The ratio of prisoners to population at the beginning of the period was 29 to 100,000; in the latter year (1904) the ratio had increased to 125. During the semi-decade, 1885-1889, there was an average of 38.5 murders and homicides to the 1,000,000 inhabitants. During a similar period, 1902-1906, the ratio had increased to 110. When we consider that such a serious crime as murder has increased threefold, the condition assumes a serious aspect. The problem is still further emphasized by the knowledge that the number of criminals in confinement at any one time compared with the total number of such offenders is small, probably not more than 10 per cent.

The highest degree of mental unbalance is found in insanity. In 1910 there were 187,791 persons confined in institutions for the insane. All but 6368 of these were in public institutions. This does not represent the entire number of insane in the country. Every community has its so-called harmlessly insane at large. Insanity, like crime and feeble-mindedness, is increasing. In a little over two decades the ratio rose from 183 per 100,000 population to 225. Statistics might be added to further illustrate the condition, but I do not wish to burden you with them.

When we consider the number of mentally deficient, the morally perverted, and the partially insane at large, their influence on social progress becomes evident. This influence is increased by the failure of our courts to protect the public against such, or to act as a deterrent force to criminals. It is further increased by the system in vogue in our penal institutions, which confirms the criminal instinct in the offender, instead of assisting in his reformation. The continuance of this symptom of the social malady means individuals and racial decadence.

The second serious symptom of the social malady may be seen in the prevalence of physical disease. While the increase in pauperism, feeble-mindedness, crime and insanity indicate a decline in the average mentality, the prevalence of bacterial diseases, infant maladies, tuberculosis, alcoholism and venereal diseases in no less degree indicate physical decadence or inefficiency, and in a degree denote our position in the scale of human evolution. At the end of a century of the most wonderful scientific advancement the world has ever known, and in which the average life has been lengthened nearly fifteen years (45), we are confronted with the fact that practically one person in every fifty lives off the illness of the other forty-nine. Pasteur, years ago, declared truthfully that it is within the power of man to rid himself of every parasitic disease. Yet at this day typhoid fever, which is due to the neglect of ordinary sanitary precaution, and the prevalence of which is taken as a criterion of sanitary inefficiency, has a death rate of 23.5 per 100,000 population (1910, 21,720), and places our sanitary efficiency along with that of Spain and Hungary (each 24). We cannot plead ignorance of sanitary matters, but the presence of bacterial diseases indicates the inability of the average mind to grasp their significance. In a city of 100,000 people there are on an average 1500 deaths from all causes each year. In cities of this size in 1910 the number of deaths actually varied from 1100 in one to 2260 in another. Let us note the part played by the bacterial diseases, 70 per cent of which we know to be preventable. Typhoid fever caused on an average 23.5 deaths. With good sanitation the number was reduced to 8.8 in one while with poor sanitation it rose to 86.7 in another. In some European cities the rate is as low as 2, while in our own army death from typhoid fever has been banished, a thing that can be done in practically every community. Measles and whooping cough combined destroy nearly 24 more (measles 12.3, whooping cough 11.6) and prepare the soil for an unknown number of cases of tuberculosis. Diphtheria, in spite of antitoxin, with the aid of unsanitary surroundings, contempt for quarantine laws and Christian Science, levies a tax of 21.4 lives annually. In our vital statistics tuberculosis is charged with 160 lives, but if the number wrongfully charged against influenza, heart disease and pneumonia, were placed in their proper account, it would far exceed 200. Despite the fact that we know the specific cause and have preached sanitation for twenty years, we have not yet perceptibly diminished its ravages. No man can estimate the tax alcoholism demands in life. The best authorities state that the venereal diseases are responsible for one-eighth of all sickness in this country. Our knowledge of these diseases, their cause, the source of infection and their effects is complete, yet very few communities have risen to an honest attempt to stamp them out. The most striking evidence of physical decadence at a time when the general intelligence is so high is seen in our startling infant mortality. Nearly one-third of a million die annually under two years of age, or 345 per 100,000. An Italian philosopher declared this was a necessary evolution. The factors that control the destiny of the child are inheritance and environment. Premature births and congenital debility destroy over 23 per cent of those dying under one year. These very conditions indicate parental disease, and the offspring came into existence too weak to stand the first shocks of life. Nearly 30 per cent of infant deaths are due to improper food—an environmental condition. In the upper strata of human society the mother too often refuses to do the mother's part. In the lower strata the mother cannot do her part. Death is the penalty exacted of the offspring. Indifference to offspring is a striking symptom of degeneracy. The maternal instinct is the strongest of all instincts.

The ideal life is one free from sickness. The ratio of those who escape more or less lengthened periods of illness to those who do not is very small. An individual who is born of healthy parents, lives a normal life and observes the simple laws of hygiene, should pass imperceptibly into old age and die a natural death, free from disease. Natural death is almost unknown in the human family today. Practically every individual dies of some disease. Disease means premature death after a greater or less period of illness. For each death there is an average period of sickness of two years. This means that the average individual is sick about one-twenty-fourth of his life. Forty-five years is the average life today. The natural life should approximate one hundred years. Life is a continuous adjustment of internal to external relations. If we are capable of such adjustment, life may be long and enjoyable. If we fail death is the penalty. In this connection what is true of the individual is already true of the race. If it cannot adapt itself to changing conditions or correct evil tendencies, destruction is inevitable.

Intimately associated with and to a large degree dependent upon its physical and mental status is the birth rate of a people. At the present time a symptom of social disorder which most students of social questions consider of the utmost importance is manifest in a declining death rate. The mere decrease in the number of births in a State or a people may be of no significance, for it may be offset by a decreasing death rate or other compensatory condition. But when a decline sets in and continues through several decades and appears unevenly distributed through the various strata of society, it may have a very grave significance. At the taking of the 1850 census there were nearly three and one-half million families. At the 1910 census there were over twenty million families. In 1850 the size of the average American family was 5.6 individuals, but in 1910 it was 4.5, a decrease of over one person to the family. The family is usually composed of parents and children, although for enumeration purposes it may contain others. This decrease means that the average family in 1910 contained one child less than the family of 1850, a decline of over 20 per cent; and, furthermore, this decline has been regularly noted in each decade of the last sixty years. This would be of little significance as affecting the national characteristics of the people if the ratio of decline appeared the same in all classes of society. The biological law that a group of organisms will tend to maintain constant characteristics through successive generations only when all parts of the group are equally fertile, applies to human society. In the early history of this country there was no such a thing as class distinction, but as the nation grew older certain broad groups appeared, indicated largely by the social standing. If the fertility of one group increase out of proportion to that of the other groups, the predominating group will tend to impress its characteristics upon the whole social structure. Pearson has pointed out that 25 per cent of the married couples produce 50 per cent of the children. Taking these points into consideration, the source of the population is always a matter of grave concern to the sociologist.

In this country our great men, statesmen, educators, scientists, engineers, financiers, etc., have come from neither the highest social rank nor the lower strata of society, but from that great middle class upon which the nation depends to maintain its social equilibrium. Changes in this great middle group occur more slowly than in either of the other groups, but even this group shows a declining birth rate. While there has been a general decline in births, the higher social ranks have been most affected, and the lower social ranks the least. This condition must in time impress the mental and moral characteristics of that class upon society. High fertility and high desirability are not always found in the same class.

In order for a nation to maintain its normal population each marriage must result in the production of four children. The average American family has already fallen below this number. According to Pearson, the family records of normal individuals show an average of 5.3 children. The intellectual classes fall far below this number, while the defective classes rise above it. Among American graduates this condition is most marked. President

Eliot in his report for 1901-1902 stated that 634 married Harvard graduates of certain years had an average family of 1.99 surviving children. It is said that the Harvard graduate has on an average three-fourths of a son, and the Vassar graduate one-half of a daughter. In 1800 the gross size of the family of the college graduate was 5.6, while in 1875 it had dwindled to 2.5 and 2. If this rate of decline persists college graduates twenty years hence will have no children. This indicates the facility with which the intellectual class eliminates itself as a factor in maintaining racial characteristics. Among American deaf-mutes, on the other hand, a highly undesirable class, the average number of children per family is 6.1. Statistics show that the mentally defective average 7 children per family, while the criminal class averages 6.6. According to statistics which are reliable, the highest fertility exists among the most undesirable, and this condition cannot otherwise in time than stamp the moral and mental characteristics of the predominating group upon the American people.

The size of the family is, in another way, a factor in maintaining the social status. The incidence of the mental and physical conditions noted heretofore have been carefully studied in reference to the number of the children of the family. The first child is twice as apt to suffer from mental and physical disabilities as the third child. The small family, on the average, contributes much more than its share towards racial destruction.

To the student of sociology these symptoms are significant of racial instability, and in a nation of national instability. The evolution of a people will follow the course indicated by the resultant of the several forces acting. Evolution, then, will be upward or downward according to the predominating factors. We are attempting to direct the course of evolution in some ways. In the earlier stages of the human race natural selection could be depended upon to eliminate a certain proportion of the unfit. But we have advanced far into a period where the survival of the fittest is supplanted by a survival of a large class which owes its very existence to the advance in sanitary science. Therefore the balance that was maintained by the more virile members is destroyed. The problems that thus arise call for other attempts to control social evolution. Our faith in our ability to meet the conditions that, uncorrected, must mean racial destruction, must rise to the social emergency. It must, however, be something more than the faith of the Turkish official who declared that "Allah wills that all shall die; some die young and some die old; but Allah wills that all shall die."

